# “No Waiting” in the “Waiting Room”: The Self-rooming Patient Pilot Study

**DOI:** 10.7759/cureus.6238

**Published:** 2019-11-26

**Authors:** Richard J Presutti, Floyd B Willis, Ruel Scott, Hope E Greig, Abd Moain Abu Dabrh

**Affiliations:** 1 Medicine, Mayo Clinic, Jacksonville, USA; 2 Family Medicine, Mayo Clinic, Jacksonville, USA

**Keywords:** waiting time, rooming, information technology, patient satisfaction, primary care

## Abstract

Introduction: Although patient timeliness and appointment flow are highly important for patients and practices, the impact of technology on improving these aspects of healthcare delivery are not widely studied. We evaluated the satisfaction and acceptability of using a handheld internet-enabled tablet computer (the Mobile Patient Communicator (MPC)) that uses interactive maps, and visual and written instructions to direct patients from waiting rooms to exam rooms independently of medical personnel.

Methods: At the time of appointment check-in, eligible patients attending their healthcare appointments at a family medicine practice received the MPC that provided them an online orientation about its use and function. The MPC directed patients to their assigned exam rooms. Patients completed pre-/post-visit surveys. We used Wilcoxon rank-sum tests for numeric variables and Fisher’s exact tests for categorical variables.

Results: Among 200 participated patients, the median level of satisfaction was 9 (1=not at all, 10= very much satisfied), 177 (91%) were successful in finding their room, and 147 (76%) thought the device should be used in the future. Prior to using the MPC, patients ≥65 years old were less comfortable with using the device (median 7 vs. 9; P=0.001), expected to have more problems operating the device (yes 6% vs. 1%; P=0.002), and were less likely to use a computer daily (yes 51% vs. 91%; P<0.001) vs. <65 years old. After using the MPC, patients ≥65 years old were less satisfied with using the device (median 8 vs. 10; P=0.001) but were more likely to watch the video on the device (yes 70% vs. 54%; P=0.04) vs. <65 years old.

Conclusion: The pilot results show evidence that using this technology for self-rooming by patients is highly acceptable regardless of age and sex. The findings also indicate this technology was helpful in delivering health care-related information before face-to-face appointments.

## Introduction

The time patients spend waiting to meet with their clinicians has been identified as one the six domains of quality by the National Academy of Medicine (NAM) (previously called the Institute of Medicine IOM) [1] as a measure of healthcare system capacity to improve. The patient’s visit to the physician’s office should be as efficient and minimally disruptive to their lives as it is therapeutic. Traditional methods of rooming patients involve a member of the healthcare team tracking the arrival and departure of patients, monitoring room availability, escorting patients to an available room, obtaining vital signs, and activating an appropriate clinician notification system to advise about patient readiness. Care team members typically have many duties apart from the rooming of patients, including answering telephone requests for results, filling prescriptions, administering medication or other treatments, and providing vaccinations, all of which often cannot be completed in the standard workday.

Over the past three decades, the healthcare sector has launched multiple initiatives that promoted the use of various technologies aimed to improve the quality of care and clinical outcomes, minimize healthcare burden on the lives of the patients, and reduce costs through novel technology-enhanced healthcare delivery models [2-4]. Many interventions aim to improve various facets of patient care including adherence to treatment, access to care and practice infrastructure support [5-7]. Using computer technology to improve the quality and delivery of care has been well recognized and continues to be explored by an increasing number of medical professionals and technology specialists [8-10]. Additionally, the use of technology has shown various impact on means of delivering health care information to patients, providing additional educational portals for patients, cost and resource utilization, increasing patient engagement, and minimizing workload on clinicians and patients [11-17]. While the relationship between timeliness and patient-centered reported outcomes in various care settings is well studied [18-20], the impact of using mobile technology to provide visit-pertinent information and facilitate patient rooming in the primary care clinic waiting rooms is scarcely reported [17].

The self-rooming patient pilot study was designed to assess the effectiveness of a wireless, hand-held device - the Mobile Patient Communicator (MPC)™ [21] - to facilitate the rooming of patients and assess this as a potential new technology which could help educate patients and improve the workflow process. We tested the feasibility, acceptability, and satisfaction with the self-use of this technology by patients. We reported patients’ experience with using this technology to find their room designated for care, and if they reported any benefits from patient education provided ahead of rooming through the same MPC device.

## Materials and methods

Participants

This study was approved by the Mayo Clinic Institutional Review Board (IRB). Five Family Medicine physicians within Family Medicine Practices at Mayo Clinic Florida participated in recruiting participants to the study over the course of four weeks. Primary care patients who were over 18 years old and were returning for follow-up appointments for non-urgent and non-acute problems were approached to participate in the study. Patients with limitations of their upper extremities were excluded from the use of the device. We excluded new patients, those with visual impairments that would make using the device difficult, or those identified as being unable to complete the tasks as determined by trained clinical desk staff. 

MPC tablet device

Each patient was given an MPC, a three-pound wireless, handheld tablet computer that contained interactive software, a barcode scanner, and a touch-screen, that offered password-protected internet and web portal exploration access. The used MPC was originated and designed by the International Medical Solutions® (IMS) [21] and customized with input from the study research team to reflect the practice mapping and include prompts and videos. Its dimensions were 228 mm x 146 mm x 25 mm. The screen was 7 inches and had a thin-film-transistor liquid-crystal display (TFT LCD) with a native resolution of 800 x 480. It had a built-in 1W speaker, 3.5 mm headphone and stereo/microphone combo jack, 4 Wire resistive touch screen, a synaptic TS42P016 stick cursor, 2 x 4-pin USB 2.0, 1x 36-pin cradle connector, 1 x DC-in, a 802.11b/g networking card with a power ON/OFF switch, Bluetooth 2.0 + EDR built-in module, 256MB to 1GB DDR2 memory, a 40GB to 160GB hard drive, and a rechargeable and replaceable lithium-ion battery. The software included is the MPC that uses an interactive map, visual and written instructions to direct patients from waiting rooms to exam rooms independently of medical personnel.

Procedure

When eligible patients arrived at the Family Medicine practice waiting room and approached the receptionist’s desk to sign in, those who met the inclusion criteria were identified by front desk attendant staff and then approached by a research assistant. Upon confirming their eligibility and obtaining their agreement and consent to participate in the study and explain the procedures of this study, participants were given a brief pre-visit paper survey to complete prior to using the MPC. The survey was used to assess expectations and comfort with using this technology prior to the hands-on experience with the device. Participants were asked to complete the survey and return it to a study assistant located in the waiting room. The study assistant then instructed patients to how to use the MPC and familiarize them with its use and functions to view the brief informational video on the tablet itself. While waiting for their exam rooms to be readied, patients viewed a brief “Welcome to the Practice” video message [13]. The MPC also provided them with optional access to various healthcare related informational and educational videos, which patients may opt out of watching and wait until their receive a message about readiness for rooming. These educational and informational videos included 20 different videos on health and wellness topics were selected from more than 100 Mayo Foundation-produced videos specifically crafted to educate patients on medical subject matter. These videos were already available to patients on desktop computers, but inaccessible to patients in waiting rooms. At the appropriate time, the MPC used flashing lights and a tone to direct patients to their exam room locations. The MPC provided an interactive map with visual and written instructions that directed patients to their assigned exam rooms. Upon arrival, patients were asked to indicate their exam room entrance by pressing a button on their MPC. A wireless signal was then sent to the patient’s physician and nurse to ensure medical personnel were aware that patients were ready for examination. At this point, the nurse would enter the room to obtain vital signs, chief complaint and perform usual functions prior to the physician arriving. Following their visit with their clinicians, patients were provided an exit or post-visit survey to complete, and then instructed to leave the MPCs on the examining room desk before they exited their exam rooms. Clinical staff, including physicians, were not present while the surveys were completed. The physicians clicked on the “reset” buttons on the MPC devices prior to leaving the exam room and a nurse retrieved the device once the patient departed. Desk attendants gave the reset MPCs to arriving patients with the same instructions.

Survey

Patients were presented self-directed pre- and post-visit surveys in paper format. The surveys were developed and revised by the study team. Patients were presented with the pre-visit survey upon checking in and were asked to rate their comfort with general use of electronic devices, affinity, and level of comfort and ease to use the MPC. Responses were collected on a scale of 0 to 10, zero being “not at all comfortable,” and 10 being “extremely comfortable.” Data on gender, age and daily computer use was also collected. The post-visit survey was completed inside the exam room upon check-out from the visit, and included questions on satisfaction, need for assistance, experience and acceptability of using the device. These responses were collected using a scale of 1 to 10, one being “not at all satisfied,” and ten being “very satisfied.” Impact on their visit experience was also collected.

Statistical analysis

Analysis of survey responses, before and after using the MPC device, were primarily descriptive in nature with the sample median, 25th percentile, and 75th percentile reported for continuous variables and number and percent reported for categorical variables. Given the small sample size, comparison of responses among participants ≥65 years vs <65 years old were explored using Wilcoxon rank-sum tests for numeric variables and Fisher’s exact tests for categorical variables. All analyses were performed using SAS statistical software (version 9.3, SAS Institute Inc.; Cary, NC).

## Results

Within the four-week active pilot time, 200 patients enrolled in this study. There were 136 (68%) participating women. The median age was 60 years (range 19-90 years). Survey responses before (pre-visit) and after (post-visit) using the MPC are summarized in Tables 1 and Table 2, respectively. The annual visit-based female-male ratio of our practice is 58.2% vs 41.8%.

**Table 1:** Pre-visit survey responses regarding use of the Mobile Patient Communicator overall and according to age

Survey item	No. of responses	Overall	Age<65 yrs. (N=117)	Age≥65 yrs. (N=82)	P value
		(N=200)			
Prior to use of the Mobile Patient Communicator					
Rate your comfort level in using this device to assist you in finding your way to your exam room (0=not at all comfortable, 10=very comfortable)	198	8 (5, 10)	9 (7, 10)	7 (5, 10)	0.001
Do you expect to be able to find your room with ease?	200				0.19
No		1 (1%)	0 (0%)	1 (1%)	
Yes		174 (87%)	105 (90%)	68 (83%)	
Don’t know		25 (13%)	12 (10%)	13 (16%)	
At this moment, do you think you will have problems operating the device?	198				0.002
No		163 (82%)	105 (90%)	57 (71%)	
Yes		6 (3%)	1 (1%)	5 (6%)	
Don’t know		29 (15%)	11 (9%)	18 (23%)	
Does the idea of using this device make you nervous at all?	198				0.42
No		181 (91%)	108 (93%)	72 (89%)	
Yes		12 (6%)	5 (4%)	7 (9%)	
Don’t know		5 (3%)	3 (3%)	2 (2%)	
Do you use the computer daily?	194				<0.001
No		49 (25%)	10 (9%)	39 (49%)	
Yes		145 (75%)	103 (91%)	41 (51%)	
How concerned are you about your appointment today? (0=not at all concerned, 10=very much concerned)	197	2 (0, 5)	1 (0, 5)	2 (0, 5)	0.33
After use of the Mobile Patient Communicator					
Rate your satisfaction with using the device. (1=not at all satisfied, 10=very much satisfied)	193	9 (7, 10)	10 (8, 10)	8 (5, 9)	<0.001
Did you require assistance from someone at the clinic to use the device or find your room?	197				0.39
No		177 (90%)	105 (92%)	71 (87%)	
Yes		18 (9%)	8 (7%)	10 (12%)	
Don’t know		2 (1%)	1 (1%)	1 (1%)	
Did you view the video on the device?	199				0.04
No		78 (39%)	53 (46%)	25 (30%)	
Yes		121 (61%)	63 (54%)	57 (70%)	
In the future, should we use this device to direct patients like you to their exam rooms?	194				0.27
No		24 (12%)	15 (13%)	9 (12%)	
Yes		147 (76%)	90 (78%)	56 (72%)	
Don’t know		23 (12%)	10 (9%)	13 (17%)	
How much of a hassle was it to use this device? (1=not at all, 10=very much a hassle)	193	1 (1, 2)	1 (1, 2)	1 (1, 3)	0.07
Is it a good idea to have patients view health information on this device prior to their appointments?	198				0.07
No		12 (6%)	7 (6%)	5 (6%)	
Yes		158 (80%)	99 (85%)	58 (73%)	
Don’t know		28 (14%)	11 (9%)	17 (21%)	
Overall, did using this device impact your experience at all today?	193				0.57
No impact		76 (39%)	42 (37%)	34 (44%)	
Positive impact		107 (55%)	65 (57%)	41 (53%)	
Negative impact		10 (5%)	7 (6%)	3 (4%)	
Data are summarized by the sample median (25^th^ percentile, 75^th^ percentile) or number (percent). Exploratory comparisons between age groups were performed using Fisher’s exact test for categorical variables and Wilcoxon rank-sum tests for numeric variables. Age was not reported for 1 patient.					

**Table 2:** Post-visit survey responses regarding use of the Mobile Patient Communicator overall and according to age

Survey item	No. of responses	Overall	Age<65 yrs. (N=117)	Age≥65 yrs.	P
		(N=200)		(N=82)	Value
After use of the Mobile Patient Communicator					
Rate your satisfaction with using the device. (1=not at all satisfied, 10=very much satisfied)	193	9 (7, 10)	10 (8, 10)	8 (5, 9)	<0.001
Did you require assistance from someone at the clinic to use the device or find your room?	197				0.39
No		177 (90%)	105 (92%)	71 (87%)	
Yes		18 (9%)	8 (7%)	10 (12%)	
Don’t know		2 (1%)	1 (1%)	1 (1%)	
Did you view the video on the device?	199				0.04
No		78 (39%)	53 (46%)	25 (30%)	
Yes		121 (61%)	63 (54%)	57 (70%)	
In the future, should we use this device to direct patients like you to their exam rooms?	194				0.27
No		24 (12%)	15 (13%)	9 (12%)	
Yes		147 (76%)	90 (78%)	56 (72%)	
Don’t know		23 (12%)	10 (9%)	13 (17%)	
How much of a hassle was it to use this device? (1=not at all, 10=very much a hassle)	193	1 (1, 2)	1 (1, 2)	1 (1, 3)	0.07
Is it a good idea to have patients view health information on this device prior to their appointments?	198				0.07
No		12 (6%)	7 (6%)	5 (6%)	
Yes		158 (80%)	99 (85%)	58 (73%)	
Don’t know		28 (14%)	11 (9%)	17 (21%)	
Overall, did using this device impact your experience at all today?	193				0.57
No impact		76 (39%)	42 (37%)	34 (44%)	
Positive impact		107 (55%)	65 (57%)	41 (53%)	
Negative impact		10 (5%)	7 (6%)	3 (4%)	
Data are summarized by the sample median (25^th^ percentile, 75^th^ percentile) or number (percent). Exploratory comparisons between age groups were performed using Fisher’s exact test for categorical variables and Wilcoxon rank-sum tests for numeric variables. Age was not reported for 1 patient.					

Prior to using the MPC, the median anticipated comfort level in using the device was 8 (0=not at all comfortable, 10=very comfortable), 87% reported that they expected to find their room with ease, 3% thought they would have problems operating the device, and 6% reported that the thought of using the device made them nervous. Daily computer use was reported by 75% of respondents, and the median concern about the appointment involving use of technology was 2 (0=not at all, 10=very much concerned).

After using the MPC, the median level of satisfaction was 9 (1=not at all satisfied, 10= very much satisfied) while only 5% reported that the device had a negative impact on their experience. Additionally, 91% were successful in finding their room while 9% reported that they needed assistance with either using the device for finding their room. Seventy-six percent thought the device should be used in the future to direct similar patients to their exam rooms, and the median for the ease of use or hassle level for using the device was 1 (1=not at all, 10=very much a hassle).

Viewing health information on the device prior to their appointment was reported to be a good idea by 80% of patients. Only 61% reported that they watched the video on the device; of these patients, 91% thought the video information was useful.

Prior to using the MPC, patients ≥65 years old tended to be less comfortable with using the device (P=0.001), expected to have more problems operating the device (P=0.002), and were less likely to use a computer daily (P<0.001) compared to patients <65 years old (Table 1). After using the MPC, patients ≥65 years old were less satisfied with using the device (P=0.001), but were more likely to watch the video on the device (P=0.04) compared to those <65 years. 

## Discussion

Our pilot study results showed that using wireless, handheld tablet medical technology for self-rooming was highly acceptable by patients regardless of age and sex. The majority of patients also reported that they found this technology helpful in delivering health care-related information before their medical appointment started while waiting.

Previous findings about patient perception and satisfaction with practices using various electronic technologies have shown mixed results [5,14,22]. For example, the use of electronic portals and telemedicine and web-based self-use technology showed generally high satisfaction by patients [2,23]. Using technology to help in monitoring or adhering to treatment has produced inconsistent results [5]. The level of acceptance of the use of this technology “after use” exceeded the “pre use” anticipated level of acceptance. This suggests that, even in a medical setting, patients are willing to welcome new technology during office visits. Though the acceptance and use of technology might vary by age [24-25], this study showed that age did not appear to be a barrier. This could be explained by the simplicity and clarity of the instruction and directions used in our MPC.

Primary care is the face of medicine to many patients receiving healthcare. Primary care clinicians tend to use more time and cost-effective approaches in treating and managing the health and wellness of their patients [26-29]. It is not surprising that primary care practices evolve and reinvent ways to face demographic and policy changes in an effort to improve patient experience and minimize the footprint of healthcare on patients’ lives. This pilot aimed to target the process of patient rooming to understand how future interventions might improve patient experience, decrease wait and disruption to their lives, and engage patients in their care while minimizing the burden on clinicians providing care to their patients.

Our study included patients that have attended our clinic while excluding new patients; therefore, this may create possible selection bias of patients familiar with the practice design and setting. This is also justified by the fact that we did not want to overwhelm new patients who are coming for the first time to establish and meet their new healthcare team with immediate involvement in research opportunities. Because of the nature of self-rooming while using MPC, this limits its use to capacitated patients. We were not able to measure how much time it took each patient to find their room. In addition, although the current iteration of the MPC effectively achieved its goals, the study provided several areas of potential improvements that would significantly improve comfort level as suggested by the patients’ responses and our experience, represented in some of the strengths of this study. These included the fact that our study is one of the first studies to describe patient satisfaction of office-based, wireless tablet-facilitated rooming of patients without the aid of a care team member. Also, the use of simple, clear and user-friendly software enhanced patient experience. The inclusion of patients of various ages and gender provides better representation to technology use and access. 

## Conclusions

Using MPC showed evidence of being an acceptable option for rooming patients and providing patient education. Our pilot findings support that such technology, if feasible, can be integrated into the work-flow process into a clinical setting, and would be accepted by most patients who are comfortable with computer use. Encouraging self-care and wellness is vitally important as we move toward value-based care and reimbursement. Potential for “in-office,” individualized delivery of health care information must be leveraged. The MPC provides this opportunity. Further research is needed on the most effective means of developing and implementing the technology of wireless, hand-held devices to improve patient and medical staff interactions and efficiency.
